# Tumors

**Published:** 1828-07

**Authors:** 


### TUMORS.

### *Cases of Tumors removed from different Parts of the Body.*

By Dr. BALLINGHALL.

I. *Warty Excrescence on the Thigh.*—JAMES CRAIG has a tumor of this description seated on the outside of the left thigh, about midway between the trochanter and condyles of the femur, and in the cicatrix of an old and extensive burn. It originally appeared in the form of a small warty excrescence about thirteen years ago; was removed by excision, and again commenced growing last spring after an injury. From this time it extended progressively in all directions, until its base occupied an extent of three or four inches diameter, in an oval or nearly circular form; the centre of it presented a foul ragged ulceration, with a fungating warty excrescence around the exterior margin: it afforded a fetid sanious discharge, and was attended with lancinating pains.

This diseased mass was removed by Dr. BALLINGALL on the 15th of October, along with a considerable part of the surrounding integument, a portion of the fascia, and of the subjacent muscular substance.

The wound left by this operation has been very slow in cicatrising, as might naturally have been expected, in consequence of the previous state of the contiguous parts from the burn; it seems for some weeks back almost stationary, with a disposition to callosity around the edges, but upon the whole exhibits a tolerably florid and healthy appearance, and the patient has left the hospital, in hopes that the change may produce a more speedy cicatrization of the sore.

The character of this affection was somewhat anomalous, and might (Dr. B. thought) be very properly included under the denomination of what has been termed warty cancer: warty in its structure, and cancerous in its disposition. He was induced to think so, from the disease having recurred in the same situation, after having been removed by Dr. Pitcairn about four years previous to the patient's admission into the hospital, and from the appearance of some enlarged glands in the groin, which seemed to be of an intractable and malignant character.

II.—Mary Goodfellow, aged sixteen, was admitted on the 6th of December, with the cuticle in various parts of the body presenting the appearance of old superficial cicatrices, apparently the result of some general cutaneous eruption; at the left commissure of the lips, at the anterior margin of the left axilla, and on the left forearm, immediately below the flexure of the elbow-joint, were prominent warty excrescences; and in the angle between the right labium pudendi and top of the corresponding thigh was another excrescence of the same character, nearly as large as a duck's egg: it was of a soft warty texture, its surface apparently consisting of numerous granular bodies, of a florid red colour; and was by many very aptly compared in its appearance to the roe of a salmon. The history given of the origin and progress of the complaint, by the patient and her mother, was exceedingly unsatisfactory, and in many respects altogether contradictory: on one occasion it was stated to have been growing from her infancy, and on another to have originated only a few months ago. By some it was considered as a form of frambœsia, or yaws; by others, as a case of sibbens; and by others, as a venereal affection. Dr. Ballingall's opinion, at first, was rather in favor of the latter supposition, chiefly from an apparent desire on the part of the patient and her mother to conceal its true origin, and from its resembling in appearance those cauliflower excrescences frequently met with on the prepuce and glans of the male, as a sequela of venereal ulcers or abrasion: at all events, the disease was obviously of an extended and constitutional character; and hence, upon consultation, it was agreed to try the effects of constitutional treatment.

The girl was therefore put upon a course of mercurial pills, and a solution of corrosive sublimate directed as a local application to the excrescence in the groin, the one in the axilla having been previously removed by a scalpel.

On the 13th of December, her mouth had become sore from the mercury, and the following report was entered in the journal:—"The swelling in the groin has much increased since her admission; a few of the largest of the small granular bodies composing the bulk of the tumor slough and fall off daily."

On the 16th, *the disease* is reported to be "still upon the increase; the angle of the mouth, the place on the anterior edge of the axilla, from which the diseased skin was removed by the knife, and two spots on the forearm, to which caustic has been applied, present the same granulated structure, only in a diminished state of activity."

On the 22d, the pills were ordered to be omitted, in consequence of the soreness of her gums; and for some days about this period she suffered considerably from febrile irritation and restlessness, with much pain from the ulcerated and sloughing points of the excrescence.

On the 5th of January, when the febrile irritation had subsided and her system appeared free from the mercurial influence, Dr. B. removed the tumor by excision, cutting out an oval portion of the integuments on which it was seated: upon dividing it longitudinally, and examining its structure, it was found that it involved the texture of the true skin.

For a few days after the operation, the patient suffered considerably from fever, her pulse being at one time as high as 134; but, on the 8th, the wound was beginning to granulate and look well; pulse eighty-two, bowels open, skin cool, and no thirst. From this time the wound continued to heal kindly, and soon cicatrised.

III.—Elizabeth Hay was admitted (11th January) for the purpose of having a tumor removed from the scalp, which is thus described in the journal: "Over the vertex of the head, and attached by a broad base, is a large firm tumor, rather greater than a clenched fist. It is moveable on the skull, and in some parts has burst, discharging a thick yellow matter around the places where it has burst; it is of a soft consistence, towards the base it feels harder. All over the body are small soft tumors, generally attached by soft pedicles, and from the size of a pea to that of a walnut.

"States that her skin has been, from her infancy, covered with these tumors, which gave her no inconvenience till within the last three years, when the one on the vertex became painful, swelled, and attained its present size. Three weeks ago it burst, and has since continued to discharge pus. General health good, bowels regular."

This tumor was removed by Dr. Hunter, and, on investigating its structure, you saw that, whatever might have been its original nature,—whether akin to the other tumors with which this patient's body was studded over, or not,—it had, previous to its removal, assumed a carcinomatous character: you saw, at some points, the appearance of fibrous bands passing through it in different directions, with matter of a dirty yellowish colour, and atheromatous consistence, occupying the interstices between them; at other points the texture of the tumor was completely broken down, and it discharged a most offensive ichorous matter, insomuch as to be loathsome to the poor woman, who earnestly entreated its removal.

In doing so, it was found that the tendon of the occipito-frontalis muscle was involved in the structure of the tumor, and the pericranium was left bare after the operation. The sore healed kindly, and the patient was dismissed cured on the 20th of February.

There were numerous tumors on this woman's body, forming a good example of the Molluscum Pendulum of BATEMAN.

IV.—Colin Wilson, aged seventeen, was admitted into the ROYAL INFIRMARY OF EDINBURGH, on the 28th of January, and the following particulars of his case entered in the journal.

"The left eye is almost protruded from, and is pushed close to the roof of the orbit; the conjunctiva palpebræ inferioris is everted, and the two eyelids cannot be brought into contact. Under the inferior palpebra, the skin is of a dusky yellow hue. Under the eye, and apparently occupying the whole of the floor of the orbit, may be felt a tumor of a soft fleshy consistence, yielding to the fingers, but not moveable. The eye is quite moveable in all directions upon the tumor; the sight is a little impaired in this eye, but he can read large print with it. Has no headache, nor pain of eye, but a sense of tension; has frequent watering of the eye, but the passage for the tears seems quite natural and unobstructed. Eight months ago received a blow on the eye, from which he suffered considerable pain: three weeks afterwards he became sensible of swelling in the orbit, which has since gradually increased, and that more rapidly of late. Leeches, blisters, and saturnine lotions have been used. A puncture was twice made with a lancet under the eyelid, from which about half an ounce of blood flowed at each time. None of these means, however, were of any advantage. Health is good. Bowels regular."

This tumor was removed by Dr. Hunter on the 31st of last month, without injury to the ball of the eye: it was soft in consistence, some portions of it quite liquid, resembling thick tar.

The swelling and symptomatic fever immediately succeeding the operation were very moderate, but at the end of about a week it was observed that the cornea was ulcerating, and this went on, notwithstanding the local application of the Nitrate of Silver and Vinum Opii, until it ended in a small protrusion of the iris, which still exists.

About the middle of the present month, this patient had a severe and obstinate attack of erysipelas, affecting the parts contiguous to the eye. This ultimately gave way to repeated bleedings ad deliquium, the internal exhibition of antimonials and saline purgatives, with the local use of anodyne fomentations.*

* Condensed from Dr. BALLINGHALL's Clinical Lectures, March 1828.

